# Crystal structure of methyl (2*Z*)-3-(4-chloro­phen­yl)-2-[(3-methyl-1*H*-indol-1-yl)meth­yl]prop-2-enoate

**DOI:** 10.1107/S2056989015010002

**Published:** 2015-05-30

**Authors:** S. Selvanayagam, B. Sridhar, S. Kathiravan, R. Raghunathan

**Affiliations:** a Department of Physics, Kings College of Engineering, Punalkulam 613 303, India; b Laboratory of X-ray Crystallography, Indian Institute of Chemical Technology, Hyderabad 500 067, India; c Department of Organic Chemistry, University of Madras, Guindy Campus, Chennai 600 025, India

**Keywords:** crystal structure, indole, methyl methacrylate, C—H⋯π inter­actions, π–π inter­actions

## Abstract

In the title indole derivative, C_20_H_18_ClNO_2_, the chloro­phenyl ring is almost perpendicular to the indole moiety, making a dihedral angle of 87.6 (1)°. The mol­ecular packing is stabilized by C—H⋯π inter­actions, which form a *C*(9) chain motif along [10-1]. In addition, there are weak π–π inter­actions [centroid–centroid distance 3.851 (1) Å] between the chains, involving inversion-related chloro­phenyl rings.

## Abstract 2

In the title indole derivative, the chloro­phenyl ring is almost perpendicular to the indole moiety, making a dihedral angle of 87.59 (6)°. In the crystal, mol­ecules are linked *via* C—H⋯π inter­actions, forming *C*(9) chains along the [10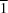] direction.

## Chemical context   

Indole derivatives inhibit hepatitis C virus replication through induction of pro-inflammatory cytokines (Lee *et al.*, 2015 ▸) and these derivatives act as a new anti-hepatitis C virus agents (Andreev *et al.*, 2015 ▸). These derivatives also act as potential mushroom tyrosinase inhibitors (Ferro *et al.*, 2015 ▸). Indole derivatives also exhibit anti-proliferative (Parrino *et al.*, 2015 ▸), anti-inflammatory (Chen *et al.*, 2015 ▸) and anti-tumor (Ma *et al.*, 2015 ▸) activities. In view of the many inter­esting applications of indole derivatives, we synthesized the title compound and report herein on its crystal structure.
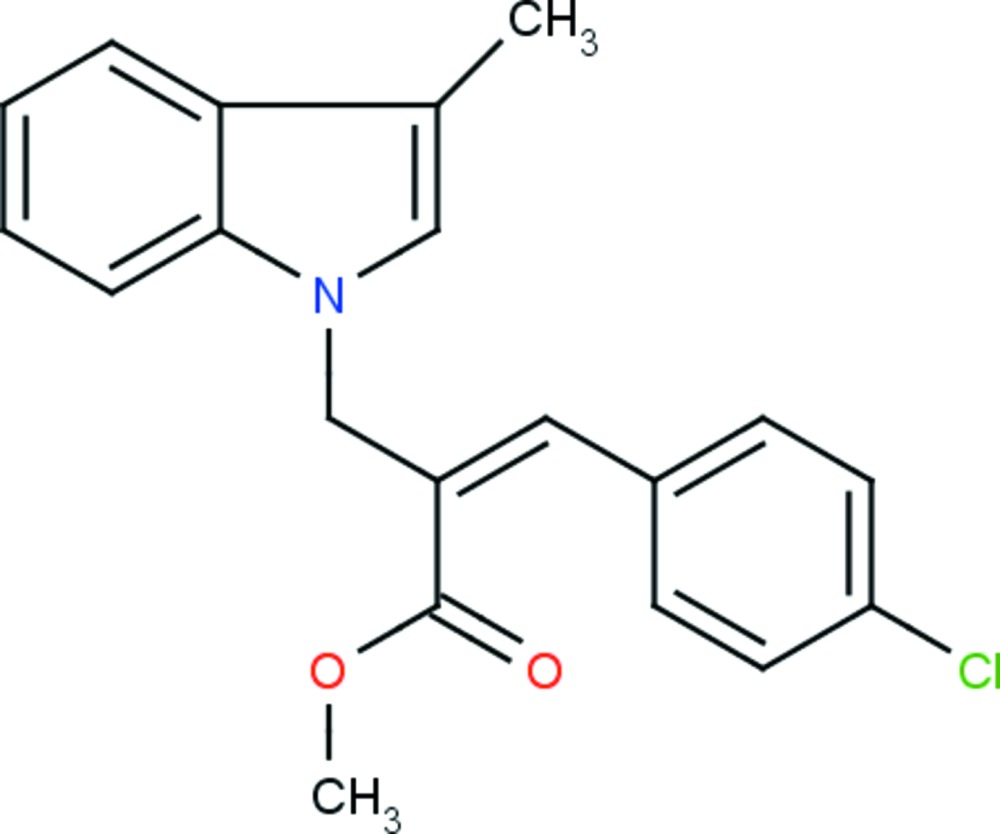


## Structural commentary   

**Figure 1:**
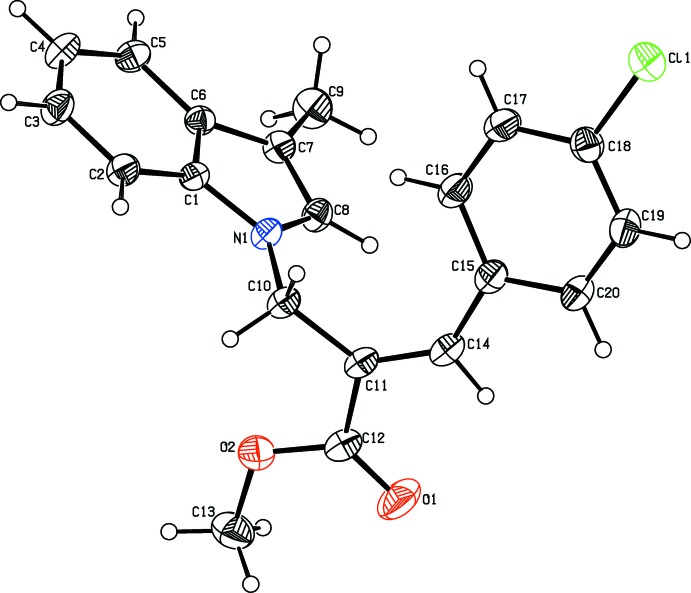
The mol­ecular structure of the title compound, showing the atom labelling. Displacement ellipsoids are drawn at the 30% probability level.

**Figure 2:**
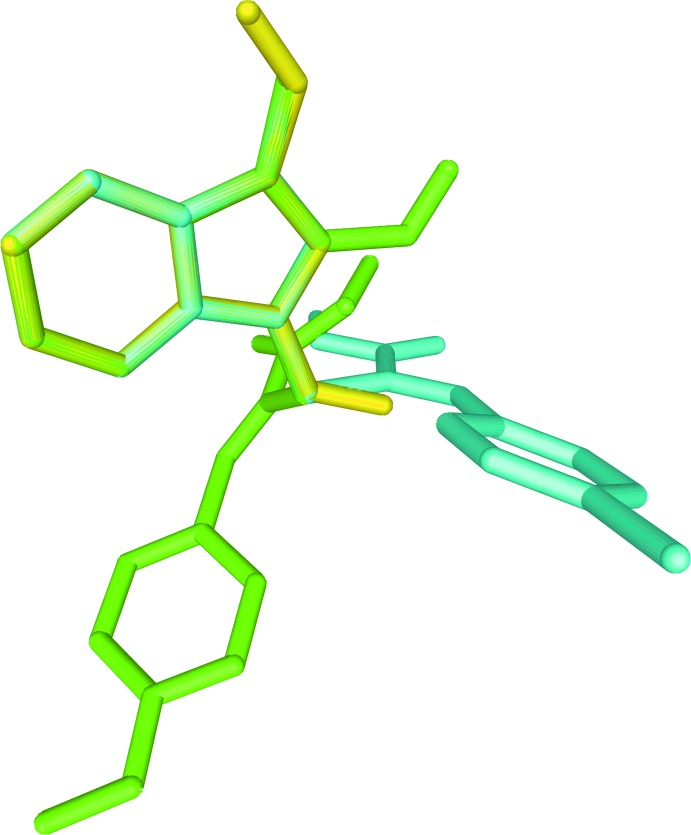
Superposition of (I) (cyan) with the similar reported structures (II) (yellow; Selvanayagam *et al.*, 2008 ▸) and (III) (green; Selvanayagam *et al.*, 2014 ▸).

The mol­ecular structure of the title compound, (I), is illus­trated in Fig. 1 ▸. The geometry of the indole ring system (N1/C1–C8) in (I) is comparable with those reported for similar structures, namely 1-vinyl-1*H*-indole-3-carbaldehyde (II) (Selvanayagam *et al.*, 2008 ▸) and methyl (2*Z*)-2-[(2-formyl-3-methyl-1*H-*indol-1-yl)meth­yl]-3-(4-meth­oxy­phen­yl)-prop-2-en­oate (III) (Selvanayagam *et al.*, 2014 ▸). The superposition of the indole ring system of (I) with the related reported structures, using *Qmol* (Gans & Shalloway, 2001 ▸), gives an r.m.s. deviation of 0.025 Å between (I) and (II), and 0.030 Å between (I) and (III); see Fig. 2 ▸. The indole ring system is planar with an r.m.s. deviation of 0.017 Å [maximum deviation of 0.028 (2) Å for atom C3], and the methyl atom C9 deviates by 0.050 (2) Å from its mean plane. The chlorine atom, Cl1, deviates by 0.008 (1) Å from the benzene ring (C15–C20) to which it is attached. This ring is almost perpendicular to the indole ring system, making a dihedral angle of 87.59 (6)°. The sum of the angles at atom N1 of the indole ring (360°) is in accordance with *sp* ^2^ hybridization. The widening of the C16—C15—C14 bond angle to 125.2 (1)° is due to the short H⋯H contact (H10*B*⋯H16 = 2.10 Å). The mean plane of the methyl methacrylate unit [O1/O2/C10–C14; maximum deviation of 0.015 (2) Å for atom O1] is almost planar with the chlrophenyl ring, making a dihedral angle of 18.98 (17)°, but is normal to the indole ring system with a dihedral angle of 89.96 (5)°.

## Supra­molecular features   

**Figure 3:**
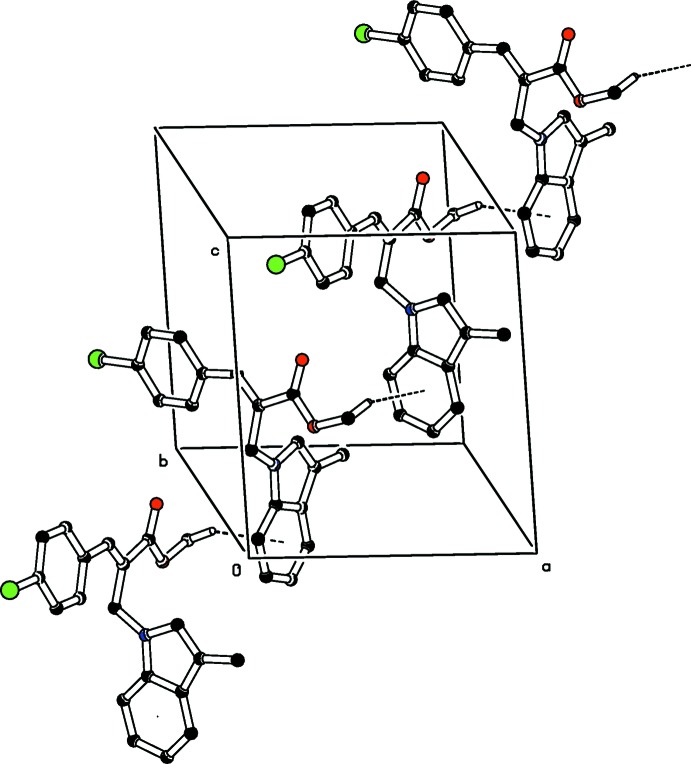
The mol­ecular packing of the title compound, viewed along the *b* axis. C—H⋯π inter­actions (Table 1 ▸) are shown as dashed lines. For clarity, H atoms not involved in these inter­actions have been omitted.

**Figure 4:**
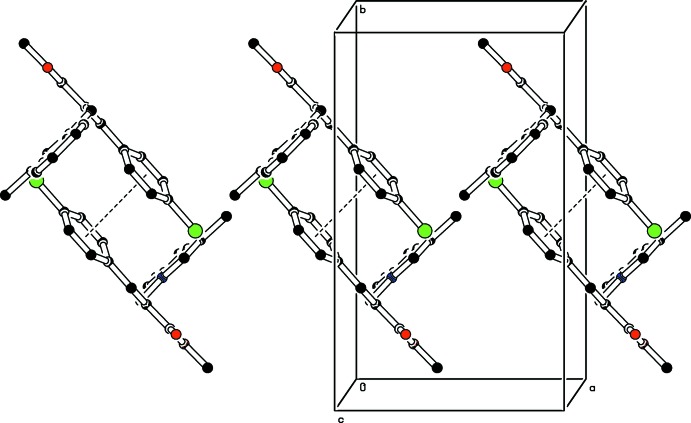
Mol­ecular packing of the title compound, showing the π–π inter­actions as dashed lines. For clarity, H atoms not involved in these inter­actions have been omitted.

In the crystal, C—H⋯π inter­actions link the mol­ecules, forming *C*(9) chains propagating along [10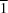]; see Fig. 3 ▸ and Table 1 ▸. Between the chains there are weak π–π inter­actions involving inversion-related chloro­phenyl rings (C15–C20), stabilizing the mol­ecular packing [centroid-to-centroid distance = 3.851 (1) Å]; see Fig. 4 ▸.

## Synthesis and crystallization   

Substituted (*Z*)-methyl-2-(bromo­meth­yl)-3-phenyl­acrylate (1 mmol), tetra-butyl-ammonium bromide (0.5 mmol), and 50% NaOH (20 ml) were added to a solution of 3-methyl indole (1 mmol) in benzene (55 ml). The mixture was stirred vigorously at room temperature for 5–6 h. The organic layer was separated, washed with water and dried over MgSO_4_. The solvent was evaporated under reduced pressure (yield: 70%). Suitable crystals were obtained by slow evaporation of a solution of the title compound in methanol at room temperature.

## Refinement   

Crystal data, data collection and structure refinement details are summarized in Table 2 ▸. H atoms were placed in idealized positions and allowed to ride on their parent atoms: C—H = 0.93–0.97 Å, with *U*_iso_(H) = 1.5*U*_eq_(C) for methyl H atoms and 1.2*U*_eq_(C) for other H atoms.

## Supplementary Material

Crystal structure: contains datablock(s) I, global. DOI: 10.1107/S2056989015010002/su5135sup1.cif

Structure factors: contains datablock(s) I. DOI: 10.1107/S2056989015010002/su5135Isup2.hkl

Supporting information file. DOI: 10.1107/S2056989015010002/su5135Isup3.cml

CCDC reference: 1402521

Additional supporting information:  crystallographic information; 3D view; checkCIF report

## Figures and Tables

**Table 1:** Hydrogen-bond geometry (, ) *Cg* is the centroid of ring C1C6.

*D*H*A*	*D*H	H*A*	*D* *A*	*D*H*A*
C13H13*A* *Cg* ^i^	0.96	2.69	3.581(2)	154

Symmetry code: (i) 
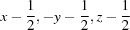
.

**Table 2:** Experimental details

Crystal data	
Chemical formula	C_20_H_18_ClNO_2_
*M*_r_	339.80
Crystal system, space group	Monoclinic, *P*2_1_/*n*
Temperature (K)	292
*a*, *b*, *c* ()	9.5867(5), 15.9077(8), 10.8902(6)
()	94.787(1)
*V* (^3^)	1654.99(15)
*Z*	4
Radiation type	Mo *K*
(mm^1^)	0.24
Crystal size (mm)	0.20 0.18 0.16

Data collection	
Diffractometer	Bruker SMART APEX CCD area detector
No. of measured, independent and observed [*I* > 2(*I*)] reflections	19078, 3944, 3313
*R*_int_	0.026
(sin /)_max_ (^1^)	0.661

Refinement	
*R*[*F* ^2^ > 2(*F* ^2^)], *wR*(*F* ^2^), *S*	0.044, 0.127, 1.02
No. of reflections	3944
No. of parameters	219
H-atom treatment	H-atom parameters constrained
_max_, _min_ (e ^3^)	0.30, 0.23

Computer programs: *SMART* and *SAINT* (Bruker, 2001 ▸), *SHELXS1997* (Sheldrick, 2008 ▸), *SHELXL2013* (Sheldrick, 2015 ▸), *ORTEP-3 for Windows* (Farrugia, 2012 ▸) and *PLATON* (Spek, 2009 ▸).

## supplementary crystallographic information

### Crystal data

**Table:**

C_20_H_18_ClNO_2_	*F*(000) = 712
*M**_r_* = 339.80	*D*_x_ = 1.364 Mg m^−^^3^
Monoclinic, *P*2_1_/*n*	Mo *K*α radiation, λ = 0.71073 Å
*a* = 9.5867 (5) Å	Cell parameters from 12437 reflections
*b* = 15.9077 (8) Å	θ = 2.3–27.7°
*c* = 10.8902 (6) Å	µ = 0.24 mm^−^^1^
β = 94.787 (1)°	*T* = 292 K
*V* = 1654.99 (15) Å^3^	Block, colourless
*Z* = 4	0.20 × 0.18 × 0.16 mm

### Data collection

**Table:**

Bruker SMART APEX CCD area-detector diffractometer	*R*_int_ = 0.026
Radiation source: fine-focus sealed tube	θ_max_ = 28.0°, θ_min_ = 2.3°
ω scans	*h* = −12→12
19078 measured reflections	*k* = −20→20
3944 independent reflections	*l* = −14→14
3313 reflections with *I* > 2σ(*I*)	

### Refinement

**Table:**

Refinement on *F*^2^	0 restraints
Least-squares matrix: full	Hydrogen site location: inferred from neighbouring sites
*R*[*F*^2^ > 2σ(*F*^2^)] = 0.044	H-atom parameters constrained
*wR*(*F*^2^) = 0.127	*w* = 1/[σ^2^(*F*_o_^2^) + (0.0722*P*)^2^ + 0.3404*P*] where *P* = (*F*_o_^2^ + 2*F*_c_^2^)/3
*S* = 1.02	(Δ/σ)_max_ = 0.002
3944 reflections	Δρ_max_ = 0.30 e Å^−^^3^
219 parameters	Δρ_min_ = −0.23 e Å^−^^3^

### Special details

**Table:**

Geometry. All esds (except the esd in the dihedral angle between two l.s. planes) are estimated using the full covariance matrix. The cell esds are taken into account individually in the estimation of esds in distances, angles and torsion angles; correlations between esds in cell parameters are only used when they are defined by crystal symmetry. An approximate (isotropic) treatment of cell esds is used for estimating esds involving l.s. planes.

### Fractional atomic coordinates and isotropic or equivalent isotropic displacement parameters (Å^2^)

**Table:**

	*x*	*y*	*z*	*U*_iso_*/*U*_eq_	
Cl1	0.85221 (5)	1.06092 (3)	0.06559 (4)	0.05976 (16)	
O1	0.23237 (16)	0.66473 (11)	−0.05769 (11)	0.0775 (4)	
O2	0.21544 (12)	0.62293 (7)	0.13579 (10)	0.0524 (3)	
N1	0.32743 (12)	0.78373 (7)	0.30864 (10)	0.0366 (3)	
C1	0.32983 (13)	0.78740 (8)	0.43477 (12)	0.0333 (3)	
C2	0.40942 (15)	0.74169 (9)	0.52459 (13)	0.0405 (3)	
H2	0.4756	0.7027	0.5035	0.049*	
C3	0.38621 (16)	0.75652 (10)	0.64588 (13)	0.0472 (4)	
H3	0.4365	0.7262	0.7077	0.057*	
C4	0.28848 (17)	0.81630 (11)	0.67783 (13)	0.0487 (4)	
H4	0.2759	0.8254	0.7605	0.058*	
C5	0.21077 (15)	0.86187 (9)	0.58952 (13)	0.0421 (3)	
H5	0.1468	0.9018	0.6120	0.051*	
C6	0.22927 (13)	0.84726 (8)	0.46513 (12)	0.0341 (3)	
C7	0.16518 (14)	0.87978 (9)	0.35139 (13)	0.0384 (3)	
C8	0.22748 (14)	0.83989 (9)	0.25968 (13)	0.0390 (3)	
H8	0.2059	0.8491	0.1759	0.047*	
C9	0.05055 (18)	0.94375 (11)	0.33648 (17)	0.0551 (4)	
H9A	0.0297	0.9559	0.2506	0.083*	
H9B	0.0800	0.9943	0.3792	0.083*	
H9C	−0.0316	0.9221	0.3701	0.083*	
C10	0.41548 (15)	0.72823 (9)	0.24128 (12)	0.0391 (3)	
H10A	0.4063	0.6711	0.2708	0.047*	
H10B	0.5126	0.7449	0.2578	0.047*	
C11	0.37749 (15)	0.73022 (9)	0.10415 (12)	0.0395 (3)	
C12	0.26941 (16)	0.67031 (10)	0.05008 (14)	0.0457 (3)	
C13	0.1091 (2)	0.56359 (11)	0.0915 (2)	0.0622 (5)	
H13A	0.0294	0.5934	0.0543	0.093*	
H13B	0.0815	0.5306	0.1592	0.093*	
H13C	0.1457	0.5272	0.0316	0.093*	
C14	0.43561 (15)	0.78067 (10)	0.02431 (13)	0.0427 (3)	
H14	0.4052	0.7706	−0.0577	0.051*	
C15	0.53784 (15)	0.84850 (9)	0.04181 (13)	0.0418 (3)	
C16	0.57356 (18)	0.89031 (11)	0.15350 (14)	0.0499 (4)	
H16	0.5315	0.8742	0.2238	0.060*	
C17	0.66983 (18)	0.95481 (11)	0.16082 (15)	0.0516 (4)	
H17	0.6930	0.9818	0.2355	0.062*	
C18	0.73146 (16)	0.97890 (9)	0.05648 (14)	0.0447 (3)	
C19	0.69899 (19)	0.93986 (11)	−0.05510 (15)	0.0521 (4)	
H19	0.7414	0.9567	−0.1248	0.063*	
C20	0.60262 (18)	0.87549 (11)	−0.06176 (14)	0.0495 (4)	
H20	0.5800	0.8492	−0.1371	0.059*	

### Atomic displacement parameters (Å^2^)

**Table:**

	*U*^11^	*U*^22^	*U*^33^	*U*^12^	*U*^13^	*U*^23^
Cl1	0.0659 (3)	0.0532 (3)	0.0606 (3)	−0.00944 (19)	0.0072 (2)	0.00371 (18)
O1	0.0809 (9)	0.1119 (12)	0.0386 (7)	−0.0357 (9)	−0.0008 (6)	−0.0147 (7)
O2	0.0572 (7)	0.0500 (6)	0.0490 (6)	−0.0075 (5)	−0.0016 (5)	−0.0016 (5)
N1	0.0384 (6)	0.0420 (6)	0.0293 (5)	0.0067 (5)	0.0016 (4)	−0.0017 (4)
C1	0.0341 (6)	0.0353 (6)	0.0304 (6)	−0.0008 (5)	0.0024 (5)	−0.0030 (5)
C2	0.0428 (7)	0.0411 (7)	0.0371 (7)	0.0080 (6)	−0.0005 (6)	−0.0006 (6)
C3	0.0524 (9)	0.0538 (9)	0.0341 (7)	0.0067 (7)	−0.0031 (6)	0.0035 (6)
C4	0.0528 (9)	0.0641 (10)	0.0292 (7)	0.0029 (7)	0.0041 (6)	−0.0049 (6)
C5	0.0407 (7)	0.0468 (8)	0.0394 (7)	0.0032 (6)	0.0070 (6)	−0.0069 (6)
C6	0.0328 (6)	0.0344 (6)	0.0351 (7)	−0.0016 (5)	0.0025 (5)	−0.0014 (5)
C7	0.0360 (7)	0.0397 (7)	0.0390 (7)	0.0028 (5)	0.0013 (5)	0.0002 (6)
C8	0.0391 (7)	0.0446 (7)	0.0324 (7)	0.0043 (6)	−0.0010 (5)	0.0031 (5)
C9	0.0504 (9)	0.0552 (10)	0.0591 (10)	0.0181 (7)	0.0013 (7)	0.0038 (8)
C10	0.0403 (7)	0.0446 (7)	0.0321 (7)	0.0069 (6)	0.0016 (5)	−0.0041 (5)
C11	0.0400 (7)	0.0478 (8)	0.0306 (7)	0.0066 (6)	0.0019 (5)	−0.0069 (6)
C12	0.0450 (8)	0.0538 (9)	0.0384 (8)	0.0033 (7)	0.0034 (6)	−0.0098 (6)
C13	0.0606 (11)	0.0505 (9)	0.0739 (12)	−0.0092 (8)	−0.0043 (9)	−0.0039 (8)
C14	0.0433 (8)	0.0540 (8)	0.0305 (7)	0.0056 (6)	0.0007 (6)	−0.0064 (6)
C15	0.0441 (8)	0.0463 (8)	0.0349 (7)	0.0072 (6)	0.0024 (6)	−0.0006 (6)
C16	0.0583 (9)	0.0570 (9)	0.0357 (8)	−0.0043 (7)	0.0108 (7)	−0.0050 (7)
C17	0.0602 (10)	0.0545 (9)	0.0406 (8)	−0.0044 (7)	0.0075 (7)	−0.0088 (7)
C18	0.0456 (8)	0.0406 (8)	0.0476 (8)	0.0049 (6)	0.0024 (6)	0.0041 (6)
C19	0.0626 (10)	0.0563 (10)	0.0384 (8)	−0.0001 (7)	0.0095 (7)	0.0067 (7)
C20	0.0606 (9)	0.0555 (9)	0.0319 (7)	−0.0002 (7)	0.0015 (6)	−0.0003 (6)

### Geometric parameters (Å, º)

**Table:**

Cl1—C18	1.7417 (16)	C9—H9B	0.9600
O1—C12	1.2007 (19)	C9—H9C	0.9600
O2—C12	1.3370 (19)	C10—C11	1.5077 (18)
O2—C13	1.442 (2)	C10—H10A	0.9700
N1—C1	1.3731 (16)	C10—H10B	0.9700
N1—C8	1.3839 (17)	C11—C14	1.338 (2)
N1—C10	1.4607 (17)	C11—C12	1.492 (2)
C1—C2	1.3940 (19)	C13—H13A	0.9600
C1—C6	1.4138 (18)	C13—H13B	0.9600
C2—C3	1.378 (2)	C13—H13C	0.9600
C2—H2	0.9300	C14—C15	1.460 (2)
C3—C4	1.399 (2)	C14—H14	0.9300
C3—H3	0.9300	C15—C20	1.400 (2)
C4—C5	1.373 (2)	C15—C16	1.403 (2)
C4—H4	0.9300	C16—C17	1.378 (2)
C5—C6	1.4001 (19)	C16—H16	0.9300
C5—H5	0.9300	C17—C18	1.378 (2)
C6—C7	1.4327 (19)	C17—H17	0.9300
C7—C8	1.363 (2)	C18—C19	1.377 (2)
C7—C9	1.497 (2)	C19—C20	1.377 (2)
C8—H8	0.9300	C19—H19	0.9300
C9—H9A	0.9600	C20—H20	0.9300
			
C12—O2—C13	116.06 (13)	N1—C10—H10B	109.1
C1—N1—C8	108.14 (11)	C11—C10—H10B	109.1
C1—N1—C10	124.46 (11)	H10A—C10—H10B	107.8
C8—N1—C10	127.39 (11)	C14—C11—C12	116.07 (13)
N1—C1—C2	129.96 (12)	C14—C11—C10	125.23 (13)
N1—C1—C6	107.92 (11)	C12—C11—C10	118.67 (13)
C2—C1—C6	122.09 (12)	O1—C12—O2	122.72 (15)
C3—C2—C1	117.42 (13)	O1—C12—C11	124.88 (16)
C3—C2—H2	121.3	O2—C12—C11	112.39 (12)
C1—C2—H2	121.3	O2—C13—H13A	109.5
C2—C3—C4	121.32 (14)	O2—C13—H13B	109.5
C2—C3—H3	119.3	H13A—C13—H13B	109.5
C4—C3—H3	119.3	O2—C13—H13C	109.5
C5—C4—C3	121.33 (13)	H13A—C13—H13C	109.5
C5—C4—H4	119.3	H13B—C13—H13C	109.5
C3—C4—H4	119.3	C11—C14—C15	132.06 (13)
C4—C5—C6	119.00 (13)	C11—C14—H14	114.0
C4—C5—H5	120.5	C15—C14—H14	114.0
C6—C5—H5	120.5	C20—C15—C16	117.42 (15)
C5—C6—C1	118.81 (12)	C20—C15—C14	117.37 (13)
C5—C6—C7	134.17 (13)	C16—C15—C14	125.19 (14)
C1—C6—C7	107.01 (11)	C17—C16—C15	121.12 (15)
C8—C7—C6	106.44 (12)	C17—C16—H16	119.4
C8—C7—C9	126.87 (14)	C15—C16—H16	119.4
C6—C7—C9	126.68 (13)	C18—C17—C16	119.37 (15)
C7—C8—N1	110.49 (12)	C18—C17—H17	120.3
C7—C8—H8	124.8	C16—C17—H17	120.3
N1—C8—H8	124.8	C19—C18—C17	121.42 (15)
C7—C9—H9A	109.5	C19—C18—Cl1	119.23 (12)
C7—C9—H9B	109.5	C17—C18—Cl1	119.34 (12)
H9A—C9—H9B	109.5	C18—C19—C20	118.89 (15)
C7—C9—H9C	109.5	C18—C19—H19	120.6
H9A—C9—H9C	109.5	C20—C19—H19	120.6
H9B—C9—H9C	109.5	C19—C20—C15	121.77 (15)
N1—C10—C11	112.50 (11)	C19—C20—H20	119.1
N1—C10—H10A	109.1	C15—C20—H20	119.1
C11—C10—H10A	109.1		
			
C8—N1—C1—C2	178.13 (14)	C8—N1—C10—C11	−6.0 (2)
C10—N1—C1—C2	−1.0 (2)	N1—C10—C11—C14	92.47 (17)
C8—N1—C1—C6	−0.08 (15)	N1—C10—C11—C12	−89.24 (16)
C10—N1—C1—C6	−179.21 (12)	C13—O2—C12—O1	0.2 (2)
N1—C1—C2—C3	−177.65 (14)	C13—O2—C12—C11	179.53 (13)
C6—C1—C2—C3	0.3 (2)	C14—C11—C12—O1	0.0 (2)
C1—C2—C3—C4	−1.2 (2)	C10—C11—C12—O1	−178.46 (16)
C2—C3—C4—C5	0.7 (3)	C14—C11—C12—O2	−179.30 (13)
C3—C4—C5—C6	0.7 (2)	C10—C11—C12—O2	2.25 (19)
C4—C5—C6—C1	−1.5 (2)	C12—C11—C14—C15	176.82 (14)
C4—C5—C6—C7	177.51 (15)	C10—C11—C14—C15	−4.9 (3)
N1—C1—C6—C5	179.37 (12)	C11—C14—C15—C20	164.77 (16)
C2—C1—C6—C5	1.0 (2)	C11—C14—C15—C16	−17.0 (3)
N1—C1—C6—C7	0.14 (15)	C20—C15—C16—C17	−0.7 (2)
C2—C1—C6—C7	−178.25 (13)	C14—C15—C16—C17	−178.87 (15)
C5—C6—C7—C8	−179.20 (15)	C15—C16—C17—C18	0.3 (3)
C1—C6—C7—C8	−0.14 (15)	C16—C17—C18—C19	0.0 (3)
C5—C6—C7—C9	−0.3 (3)	C16—C17—C18—Cl1	179.55 (13)
C1—C6—C7—C9	178.81 (15)	C17—C18—C19—C20	0.1 (3)
C6—C7—C8—N1	0.09 (16)	Cl1—C18—C19—C20	−179.51 (13)
C9—C7—C8—N1	−178.86 (14)	C18—C19—C20—C15	−0.4 (3)
C1—N1—C8—C7	0.00 (16)	C16—C15—C20—C19	0.7 (2)
C10—N1—C8—C7	179.09 (13)	C14—C15—C20—C19	179.06 (15)
C1—N1—C10—C11	172.92 (13)		

### Hydrogen-bond geometry (Å, º)

Cg is the centroid of ring C1–C6.

**Table:**

*D*—H···*A*	*D*—H	H···*A*	*D*···*A*	*D*—H···*A*
C13—H13*A*···*Cg*^i^	0.96	2.69	3.581 (2)	154

Symmetry code: (i) *x*−1/2, −*y*−1/2, *z*−1/2.
